# Coffee, Tea, and Mammographic Breast Density in Premenopausal Women

**DOI:** 10.3390/nu13113852

**Published:** 2021-10-28

**Authors:** Adashi Margaret Odama, Valerie Otti, Shuai Xu, Olamide Adebayo, Adetunji T. Toriola

**Affiliations:** 1 Division of Public Health Sciences, Department of Surgery, Washington University School of Medicine, 660 South Euclid Avenue, Campus Box 8100, St. Louis, MO 63110, USA; a.odama@wustl.edu (A.M.O.); votti@uchicago.edu (V.O.); shuaixu@wustl.edu (S.X.); olaadebayo@wustl.edu (O.A.); 2 Alvin J. Siteman Cancer Center at Barnes-Jewish Hospital and Washington University School of Medicine, St. Louis, MO 63110, USA

**Keywords:** coffee, tea, mammographic breast density, premenopausal women, breast cancer

## Abstract

Studies have investigated the associations of coffee and tea with mammographic breast density (MBD) in premenopausal women with inconsistent results. We analyzed data from 375 premenopausal women who attended a screening mammogram at Washington University School of Medicine, St. Louis, MO in 2016, and stratified the analyses by race (non-Hispanic White (NHW) vs. Black/African American). Participants self-reported the number of servings of coffee, caffeinated tea, and decaffeinated tea they consumed. Volpara software was used to determine volumetric percent density (VPD), dense volume (DV), and non-dense volume (NDV). We used generalized linear regression models to quantify the associations of coffee and tea intake with MBD measures. Coffee: ≥1 time/day (β = 1.06; 95% CI = 0.93–1.21; *p*-trend = 0.61) and caffeinated tea: ≥1 time/day (β = 1.01; 95% CI = 0.88–1.17; *p*-trend = 0.61) were not associated with VPD. Decaffeinated tea (≥1 time/week) was positively associated with VPD in NHW women (β = 1.22; 95% CI = 1.06–1.39) but not in African American women (β = 0.93; 95% CI = 0.73–1.17; *p*-interaction = 0.02). Coffee (≥1 time/day) was positively associated with DV in African American women (β = 1.52; 95% CI = 1.11–2.07) but not in NHW women (β = 1.10; 95% CI = 0.95–1.29; *p*-interaction = 0.02). Our findings do not support associations of coffee and caffeinated tea intake with VPD in premenopausal women. Positive associations of decaffeinated tea with VPD, with suggestions of effect modification by race, require confirmation in larger studies with diverse study populations.

## 1. Introduction

Mammographic breast density (MBD), the amount of epithelial and stromal tissues in relation to adipose tissue in the breast, is a risk factor for breast cancer [1,2,3,4]. Studies have reported a 4–6-fold increased risk for breast cancer among women in the highest quartile of MBD (>75%) compared to those in the lowest quartile (<25%) [5], especially in premenopausal women [4,6]. To have a greater understanding of the determinants of breast density, especially those that can be modified, studies have investigated the associations of adult diet and dietary factors with MBD. Results from those studies have, however, been largely null [7,8,9,10].

Coffee and tea are promising dietary factors to evaluate in relation to MBD because they contain phytochemicals [11] and have also been shown to influence estrogen levels and estrogen metabolites [12]. They also contain phenolic compounds, which have anti-oxidative properties [13]. Caffeine, a major component of coffee and tea, is a natural purine alkaloid that is thought to induce anticancer properties through various mechanisms including its effect on apoptosis, DNA repair capacity, and altered sex hormone levels [14,15,16].

Only a limited number of studies (four to date) have investigated the associations of coffee or tea intake with MBD, and their findings have been inconsistent [17,18,19,20]. Two studies investigated the associations between caffeine intake (including coffee) and MBD: one in premenopausal women alone [20] and the other in both pre- and postmenopausal women [19]. Both studies reported no associations between regular coffee and MBD in premenopausal women but one study observed a positive association between decaffeinated coffee and MBD [19]. Likewise, two studies have investigated the associations of tea (green and black) with MBD: one study in both pre- and postmenopausal women [17], and the other study was a randomized controlled trial (RCT) of green tea extract (GTE) supplementation on MBD in postmenopausal women [18]. The first study found an inverse association between green tea and MBD in both pre- and postmenopausal women, but the findings were not significant in premenopausal women [17]. The study also found no associations between black tea and MBD. All four studies used area-based measures of MBD and did not investigate whether associations differed by race. Our objective in this study was to investigate the associations of coffee and tea intake with MBD in premenopausal women using volumetric measures of MBD. Further, we provide the first data on the associations of coffee and tea intake with MBD in African American women.

## 2. Methods

### 2.1. Participants

We recruited 375 cancer-free premenopausal women who were scheduled for an annual screening mammogram at the Joanne Knight Breast Health Center (BHC) at Siteman Cancer Center at Washington University School of Medicine, St. Louis, Missouri in 2016. Premenopausal women scheduled for their annual screening were mailed flyers about the study in advance of their appointment. Follow-up calls were then made within seven days of the scheduled appointments to further screen interested women and clarify any concerns regarding the study. Eligibility criteria included: (i) Premenopausal at the time of mammogram. Women were identified as premenopausal if they had a regular menstrual period within the preceding 12 months, no prior history of bilateral oophorectomy, and had not used menopausal hormone therapy; (ii) no serious medical condition that would prevent the participant from returning for her annual mammogram in 12 months; (iii) not pregnant; (iv) no history of any cancer, including breast cancer; and (v) no history of breast augmentation or reduction [21]. Participants provided informed consent and study approval was granted by the Institutional Review Board (IRB) of the Washington University School of Medicine.

### 2.2. Coffee/Tea Intake

On the day of their screening, mammogram participants completed a questionnaire on several potential risk factors including coffee and tea intake.

Participants were asked how many servings of the following beverages they consume: coffee, caffeinated tea, herbal tea, or decaffeinated tea. A serving of these beverages was defined as an 8 oz glass. Coffee intake was categorized into: (i) less than once/week, (ii) 1 time/week, (iii) 2–6 times/week, (iv) 1 time/day; >1 time/day. Due to low consumption, we recategorized caffeinated tea and decaffeinated tea based on the distribution in our study population. Caffeinated tea was recategorized into: (i) less than once per week, (ii) once/week, (iii) 2–6 times/week, (iv) ≥1 time/day. Decaffeinated tea intake was recategorized into (i) <1 time/week and (ii) ≥1 time/week.

### 2.3. Mammographic Breast Density Measures

We used Volpara [version 1.5, (Matakina Technology Ltd., Wellington, New Zealand)] to determine the volumetric measures of MBD. Volpara uses a computerized algorithm that calculates the X-ray attenuation and measured breast thickness at each pixel to estimate volumetric measures [22]. Volpara volumetric percent density (VPD) measures correspond to the Breast Imaging Reporting and Data System (BI-RADS) categorical terms (5th edition). These translate to a: <3.5% (almost entirely fatty), b: ≥3.5 and <7.5% (scattered areas of fibroglandular density), c: ≥7.5 and <15.5% (heterogeneously dense), and d: ≥15.5% (extremely dense) [23].

### 2.4. Statistical Analysis

We calculated mean and standard deviations for continuous variables and percentages for categorical variables. MBD measures (volumetric percent density, dense volume, and non-dense volume) were skewed; hence, we natural log transformed them to meet the assumption of normality of the residuals. We built linear regression models to determine the associations of coffee/tea intake and log transformed the MBD measures. All models were adjusted for age (continuous), age at menarche (continuous), body mass index (continuous), parity, and age at first birth (nulliparous, 1–2 children and <25 years at first birth, 1–2 children and 25–29 years at first birth, 1–2 children and ≥30 years at first birth, ≥3 children and <25 years at first birth, ≥3 children and ≥25 years at first birth), race (NHW, African American, others), and family history of breast cancer in a first-degree relative (no, yes, missing) based on their significance at a *p*-value < 0.10 in our models. We computed back-transformed beta coefficients [exp(β)] and 95% confidence intervals from the regression models. To make the interpreting of our results easier, we presented the coefficients as percentage difference (%) [Diff % = (exp(β) − 1) ∗ 100]. Tests for trends across coffee/tea intake were performed using the Wald statistic by including the medians of each category as continuous variables in the adjusted linear models. We also used Wald tests to examine whether the associations of coffee/tea intake on MBD differed depending on race (NHW and African American). We performed a sensitivity analysis to examine the interaction between coffee/tea intake and race (NHW, African American, and Others). We conducted a subgroup analysis only for NHW and African Americans because of the small sample size of the other races (*n* = 19). All the analyses were conducted using SAS version 9.4 (SAS Institute, Cary, NC, USA). All tests were two-sided, and *p* < 0.05 was considered as the statistical significance.

## 3. Results

Of the 375 premenopausal women enrolled in our study, 65.6% were NHW and 29.3% were Black/African American (Table 1). The mean age was 47.5 years (range 32–58 years). The mean age at menarche was 12.8 years (range 9–18 years). The mean body mass index (BMI) at enrollment was 30.6 kg/m^2^. As many as 24.5% of participants drank coffee >1 time/day, 16.3% drank caffeinated tea ≥1 time/day, and 25.6% drank decaffeinated tea ≥1 time/week. The mean volumetric percent density (VPD), dense volume (DV), and non-dense volume (NDV) were 9.5%, 80.7 cm^3^, and 1079 cm^3^, respectively.

In multivariable-adjusted models, there were no associations between coffee, caffeinated tea, and VPD (Table 2). Women who drank coffee and caffeinated tea ≥1 time/day had a 6% (β = 1.06; 95% CI = 0.93–1.21; *p*-trend = 0.61) and a 1% (β = 1.01; 95% CI = 0.88–1.17; *p*-trend = 0.61) higher VPD compared to those who drank <1 time/week, respectively. Women who drank decaffeinated tea ≥1 time/week had an 11% higher VPD (β = 1.11; 95% CI = 1.00–1.25) compared to those with an intake of <1 time/week (Table 2).

We observed a positive association between coffee intake and DV (Table 3). Women who drank coffee ≥1 time/day had a 13% higher DV (β = 1.13; 95% CI = 1.01–1.29; *p*-trend = 0.05) compared to those who drank coffee <1 time/week. Caffeinated and decaffeinated tea were not associated with DV. Likewise, there were no associations of coffee, caffeinated tea, and decaffeinated tea with NDV (Table 3).

Race appears to modify the associations of coffee and tea intake with MBD. Decaffeinated tea was positively associated with VPD in NHW women but not in African American women (Table 4). NHW women with an intake of ≥1 time/week had a 22% higher VPD (β = 1.22; 95% CI = 1.06–1.39; *p*-interaction = 0.02) compared to those with an intake of <1 time/week.

Coffee intake was positively associated with dense volume (β = 1.52; 95% CI = 1.11–2.07) in African American women but not in NHW women (β = 1.10; 95% CI = 0.95–1.29; *p*-interaction = 0.02) (Table 5). Decaffeinated tea was inversely associated with NDV in NHW women (β = 0.81; 95% CI = 0.70–0.93) but not African American women (β = 1.15; 95% CI = 0.89–1.48; *p*-interaction = 0.01) (Table 5).

## 4. Discussion

We observed no associations of coffee and caffeinated tea with VPD in premenopausal women. Decaffeinated tea, however, was positively associated with VPD. We report, for the first time: possible effect modification by race on the associations of coffee and tea intake with MBD, positive associations of decaffeinated tea with VPD in NHW only, and positive associations of coffee with DV in African American only, which require confirmation in other studies.

Two studies within the Nurse Health Study (NHS) cohorts have investigated the associations of coffee and MBD in premenopausal women [19,20]. They reported no associations between adolescent coffee intake [20] and regular coffee intake in adulthood [19] with percent mammographic density (PMD) in premenopausal women, although one of them observed an inverse association of regular coffee intake in adulthood with PMD in postmenopausal women [19]. Similarly, we found no associations between coffee and VPD, even after stratifying by race. Interestingly, one of the studies reported a positive association between decaffeinated coffee and PMD in premenopausal women [19]. Although we did not include decaffeinated coffee in our analyses, we observed a similar association between decaffeinated tea and VPD. Decaffeinated tea was positively associated with VPD in NHW women but not African American women. Likewise, the NHS study population is predominantly NHW (97%) [19]. Coffee and tea are different types of beverages, and it is unclear why they should be positively associated with MBD when decaffeinated; however, our results and those from the NHS suggest possible positive associations between decaffeinated beverages and MBD in premenopausal NHW women.

We observed no associations between caffeinated tea and VPD. Our results differ from the other two studies [17,18] that investigated associations between tea and MBD. In the first study with 2859 Chinese women, daily green tea drinkers had a 2.2% lower PMD when compared to non-tea drinkers, while black tea was not associated with PMD [17]. After stratifying the results by menopausal status, the inverse association between green tea and PMD remained in premenopausal women but was not statistically significant [17]. The second study was an RCT that observed no differences in PMD between the placebo group and the GTE group after one-year of GTE supplementation. However, after stratifying by age, they observed that GTE was associated with a 4.4% reduction in PMD in the youngest age group (50–55 years), although the interaction between age and GTE on PMD was only borderline statistically significant [18]. The differences in our results, when compared to these studies, might be due to various factors: Participants in the RCT were postmenopausal (mean age was 60 years) [18], and while participants in the Chinese study were both pre- and postmenopausal, they were older than 50 years [17]. In contrast, our study was limited to premenopausal women, and our study population was younger with a mean age of 47.5 years. Both studies focused on specific types of tea: the Chinese study on green and black tea, and the RCT study on green tea extract. However, we evaluated caffeinated and decaffeinated tea, not a specific tea type (green or black tea). Further, participants in the first study were Chinese [17] while the majority of the women (>97%) in the RCT study were NHW women [18]. In our study, close to 30% of our participants were African American.

To the best of our knowledge, our study is the first to evaluate the associations of coffee and tea intake with MBD in African American women. We observed that decaffeinated tea was positively associated with VPD in NHW women but not in African American women. On the other hand, coffee was positively associated with DV in African American women but not in NHW women. Given these findings, larger studies with diverse populations are needed to investigate whether race modifies the associations of coffee and tea with MBD.

Coffee and tea can impact estrogen levels and estrogen metabolites [12], and they contain phytochemicals through which they can impact cell proliferation and differentiation [11]; hence, their associations with MBD are biologically plausible. Further, caffeine, one of the main components of coffee and tea, inhibits protein kinases such as the ataxia-telangiectasia mutated (ATM)/Rad3-related (ATR) kinases as well as induces anticancer potential through apoptosis [14]. Some tea catechins have also been found to have inhibitory effects on breast cancer [24]; this observation has mostly been in relation to green tea [25,26,27], while black tea has been found to have positive associations with breast cancer [28,29,30].

Studies have reported possible associations between some diet and dietary factors, such as dairy products (including milk), adolescent intake of animal fat, and olive oil with MBD [31,32,33]. Given that diet is modifiable, there is the need for more studies looking at the associations of diet and dietary factors, as well as their interactions with MBD.

Our study has some limitations: It is cross-sectional. Information on coffee and tea intake was self-reported by participants, but we do not expect that there will be a differential recall of coffee or tea intake based on MBD. Although we adjusted for potential confounders, residual confounding might remain.

Despite these limitations, our study has the following strengths: Study participants were recruited from all women attending annual routine screening mammography at the Joanne Knight Breast Health Center, improving generalizability. We assessed MBD using Volpara, which provides volumetric measures of density and has been shown to provide highly reproducible results [34,35].

## Figures and Tables

**Table 1:** Characteristics of 375 premenopausal women recruited during the annual screening mammogram.

Characteristic	Number	Mean ± SD ^a^/Percentage
Age, years	375	47.5 ± 4.8
Age at Menarche, years	373	12.8 ± 2.2
Body Mass Index (BMI), kg/m^2^	375	30.8 ± 8.1
Family history of breast cancer		
Yes	88	23.5%
No	287	76.5%
Race		
NHW	246	65.6%
Black/African American	110	29.3%
Others/Unknown	19	5.1%
Coffee intake		
<1 time/week	133	35.5%
1 time/week	22	5.9%
2–6 times/week	37	9.9%
1 time/day	85	22.7%
≥1 time/day	92	24.5%
Missing	6	1.6%
Caffeinated tea intake		
<1 time/week	161	42.9%
1 time/week	63	16.8%
2–6 times/week	71	18.9%
≥1 time/day	61	16.3%
Missing	19	5.1%
Decaffeinated tea intake		
<1 time/week	250	66.7%
≥1 time/week	96	25.6%
Missing	29	7.7%
Parity and age at first birth		
Nulliparous	70	18.7%
1–2 children, <25 years	65	17.3%
1–2 children, 25–29 years	61	16.3%
1–2 children, ≥30 years	79	21.1%
≥3 children, <25 years	61	16.3%
≥3 children, ≥25 years	36	9.6%
Missing	3	0.8%
Mammographic breast density		
Volumetric Percent Density (%)	375	9.5 ± 6.5
Non-Dense Volume (cm^3^)	375	1079.0 ± 743.0
Dense Volume (cm^3^)	375	80.7 ± 42.7

^a^ SD = standard deviation.

**Table 2:** Associations of coffee/tea intake with volumetric percent density in premenopausal women *.

Frequency	Number	exp(β) (95% CI)	*p*-Trend
Coffee			0.61
<1 time/week	133	Ref	
1 time/week	22	1.02 (0.82, 1.26)	
2–6 times/week	37	0.93 (0.78, 1.11)	
1 time/day	85	0.98 (0.86, 1.13)	
≥1 time/day	92	1.06 (0.93, 1.21)	
Caffeinated tea			0.61
<1 time/week	161	Ref	
1 time/week	63	0.97 (0.84, 1.11)	
2–6 times/week	71	0.90 (0.78, 1.03)	
≥1 time/day	61	1.01 (0.88, 1.17)	
Decaffeinated tea			-
<1 time/week	250	Ref	
≥1 time/week	96	1.11 (1.00, 1.25)	

* Adjusted for age (continuous), body mass index (continuous), family history of breast cancer in a first-degree relative (no, yes), race (NHW, African American, others), age at menarche (continuous), parity and age at first birth (nulliparous, 1–2 children and <25 years at first birth, 1–2 children and 25–29 years at first birth, 1–2 children and ≥30 years at first birth, ≥3 children and <25 years at first birth, ≥3 children and ≥25 years at first birth); CI = confidence intervals, Ref = reference group, exp(β) = back-transformed beta coefficient.

**Table 3:** Associations of coffee/tea intake with dense volume and non-dense volume in premenopausal women *.

Frequency	Number	Dense Volume		Non-Dense Volume	
		exp(β) (95% CI)	*p*-Trend	exp(β) (95% CI)	*p*-Trend
Coffee			**0.05** ^a^		0.34
<1 time/week	133	Ref		Ref	
1 time/week	22	0.97 (0.79, 1.19)		0.96 (0.76, 1.20)	
2–6 times/week	37	1.06 (0.90, 1.24)		1.14 (0.95, 1.38)	
1 time/day	85	1.06 (0.94, 1.20)		1.03 (0.90, 1.19)	
≥1 time/day	92	**1.13 (1.01, 1.29)**		1.06 (0.92, 1.23)	
Caffeinated tea			0.53		0.75
<1 time/week	161	Ref		Ref	
1 time/week	63	0.92 (0.81, 1.05)		0.98 (0.84, 1.14)	
2–6 times/week	71	0.93 (0.82, 1.05)		1.06 (0.92, 1.22)	
≥ 1 time/day	61	0.99 (0.87, 1.12)		0.99 (0.85, 1.16)	
Decaffeinated tea			-		-
<1 time/week	250	Ref		Ref	
≥ 1 time/week	96	1.03 (0.93, 1.15)		0.88 (0.78, 1.00)	

* Adjusted for age (continuous), body mass index (continuous), family history of breast cancer in a first-degree relative (no, yes), race (NHW, African American, others), age at menarche (continuous), parity and age at first birth (nulliparous, 1–2 children and <25 years at first birth, 1–2 children and 25–29 years at first birth, 1–2 children and ≥30 years at first birth, ≥3 children and <25 years at first birth, ≥3 children and ≥25 years at first birth); significant coefficients and *p* trend values are in bold print. CI = confidence intervals, Ref = reference group, exp(β) = back-transformed beta coefficient. ^a^ *p*-trend value is 0.049.

**Table 4:** Associations of coffee/tea intake with volumetric percent density stratified by race *.

Frequency	NHW			African American			*p*-Interaction ^a^
	Number	exp(β) (95% CI)	*p*-Trend	Number	exp(β) (95% CI)	*p*-Trend	
Coffee			0.86			0.77	0.30
<1 time/week	75	Ref		52	Ref		
1 time/week	5	0.79 (0.51, 1.24)		14	1.11 (0.86, 1.44)		
2–6 times/week	20	1.02 (0.80, 1.29)		17	0.83 (0.65, 1.05)		
1 time/day	62	0.96 (0.81, 1.13)		18	0.98 (0.77, 1.26)		
≥1 time/day	79	1.02 (0.87, 1.20)		8	1.10 (0.79, 1.55)		
Caffeinated tea			0.57			0.38	0.06
<1 time/week	101	Ref		55	Ref		
1 time/week	42	1.04 (0.87, 1.24)		16	0.71 (0.55, 0.91)		
2–6 times/week	48	0.96 (0.81, 1.13)		22	0.82 (0.66, 1.01)		
≥1 time/day	42	0.97 (0.81, 1.15)		12	0.90 (0.79, 1.38)		
Decaffeinated tea			-				**0.02**
<1 time/week	157	Ref		84	Ref	-	
≥1 time/week	71	**1.22 (1.06, 1.39)**		19	0.93 (0.73, 1.17)		

* Adjusted for age (continuous), body mass index (continuous), family history of breast cancer in a first-degree relative (no, yes), parity and age at first birth (nulliparous, 1–2 children and <25 years at first birth, 1–2 children and 25–29 years at first birth, 1–2 children and ≥30 years at first birth, ≥3 children and <25 years at first birth, ≥3 children and ≥25 years at first birth); significant coefficients and *p* trend values are in bold print. CI = confidence intervals, Ref = reference group, exp(β) = back-transformed beta coefficient. ^a^ Testing for interaction for race (NHW and African American) and coffee/tea intake was performed based on the Wald test.

**Table 5:** Associations of coffee/tea intake with dense volume and non-dense volume stratified by race *.

	Dense Volume							Non-dense Volume						
	NHW			African American			*p*-Int ^a^	NHW			African American			*p*-Int ^a^
	N	exp(β) (95% CI)	*p*-trend	N	exp(β) (95% CI)	*p*-Trend		N	exp(β) (95% CI)	*p*-Trend	N	exp(β) (95% CI)	*p*-Trend	
Coffee			0.21			**0.04**	**0.02**			0.49			**0.05** ^b^	0.25
<1 time/week	75	Ref		52	Ref			75	Ref		52	Ref		
1 time/week	5	0.91 (0.60, 1.39)		14	0.99 (0.78, 1.26)			5	1.17 (0.73, 1.86)		14	0.87 (0.66, 1.17)		
2–6 times/week	20	1.25 (0.99, 1.57)		17	0.90 (0.72, 1.11)			20	1.23 (0.96, 1.58)		17	1.11 (0.85, 1.44)		
1 time/day	62	1.05 (0.90, 1.23)		18	1.17 (0.94, 1.46)			62	1.03 (0.87, 1.23)		18	1.18 (0.90, 1.54)		
≥1 time/day	79	1.10 (0.95, 1.29)		8	**1.52 (1.11, 2.07)**			79	1.07 (0.91, 1.27)		8	1.39 (0.96, 2.01)		
Caffeinated tea			0.79			0.50	0.92			0.40			0.75	0.14
<1 time/week	101	Ref		55	Ref			101	Ref		55	Ref		
1 time/week	42	0.98 (0.82, 1.16)		16	0.83 (0.66, 1.04)			42	0.97 (0.80, 1.17)		16	1.21 (0.91, 1.61)		
2–6 times/week	48	0.95 (0.81, 1.12)		22	0.94 (0.77, 1.14)			48	1.03 (0.86, 1.24)		22	1.17 (0.92, 1.49)		
≥ 1 time/day	42	0.99 (0.84, 1.18)		12	0.95 (0.73, 2.07)			42	1.08 (0.90, 1.31)		12	0.90 (0.65, 1.24)		
Decaffeinated tea						-	0.98			-			-	**0.01**
<1 time/week	157	Ref		84	Ref			157	Ref		84	Ref		
≥1 time/week	71	1.05 (0.92, 1.20)		19	1.06 (0.86, 1.31)			71	**0.81 (0.70, 0.93)**		19	1.15 (0.89, 1.48)		

* Adjusted for age (continuous), body mass index (continuous), family history of breast cancer in a first-degree relative (no, yes), parity and age at first birth (nulliparous, 1–2 children and <25 years at first birth, 1–2 children and 25–29 years at first birth, 1–2 children and ≥30 years at first birth, ≥3 children and <25 years at first birth, ≥3 children and ≥25 years at first birth); significant coefficients and *p* trend values are in bold print. N = number, CI = confidence intervals, Ref = reference group, exp(β) = back-transformed beta coefficient. ^a^ *p*-int = *p*-interaction. Testing for interaction for race (NHW and African American) and coffee/tea intake was based on the Wald test. ^b^ *p*-trend value is 0.047.

## Data Availability

The original contributions presented in the study are included in the article, further inquiries can be directed to the corresponding authors.
